# CORAL Models for Drug-Induced Nephrotoxicity

**DOI:** 10.3390/toxics11040293

**Published:** 2023-03-23

**Authors:** Andrey A. Toropov, Devon A. Barnes, Alla P. Toropova, Alessandra Roncaglioni, Alasdair R. Irvine, Rosalinde Masereeuw, Emilio Benfenati

**Affiliations:** 1 Istituto di Ricerche Farmacologiche Mario Negri IRCCS, Via Mario Negri 2, 20156 Milano, Italy; alla.toropova@marionegri.it (A.P.T.); alessandra.roncaglioni@marionegri.it (A.R.); emilio.benfenati@marionegri.it (E.B.); 2 Utrecht Institute for Pharmaceutical Sciences, div. Pharmacology, Universiteitsweg 99, 3584 CG Utrecht, The Netherlands; d.a.barnes@uu.nl (D.A.B.); a.r.irvine@uu.nl (A.R.I.); r.masereeuw@uu.nl (R.M.)

**Keywords:** SAR, nephrotoxicity, Monte Carlo method, semi-correlation, CORAL software

## Abstract

Drug-induced nephrotoxicity is a major cause of kidney dysfunction with potentially fatal consequences. The poor prediction of clinical responses based on preclinical research hampers the development of new pharmaceuticals. This emphasises the need for new methods for earlier and more accurate diagnosis to avoid drug-induced kidney injuries. Computational predictions of drug-induced nephrotoxicity are an attractive approach to facilitate such an assessment and such models could serve as robust and reliable replacements for animal testing. To provide the chemical information for computational prediction, we used the convenient and common SMILES format. We examined several versions of so-called optimal SMILES-based descriptors. We obtained the highest statistical values, considering the specificity, sensitivity and accuracy of the prediction, by applying recently suggested atoms pairs proportions vectors and the index of ideality of correlation, which is a special statistical measure of the predictive potential. Implementation of this tool in the drug development process might lead to safer drugs in the future.

## 1. Introduction

Nephrotoxicity refers to the harmful effects that occur in the kidneys due to chemicals and medicines, known as nephrotoxicants, often resulting in their rapid deterioration. The kidneys are uniquely susceptible to drug-induced injury due to their high cardiac output and their role in the excretion of waste compounds from the body. Due to their pivotal role in concentrating and reabsorbing the glomerular filtrate, the kidney proximal tubular cells are particularly prone to elevated levels of circulating toxicants. Drug-induced nephrotoxicity (DIN) has been identified as a major contributor to both acute kidney injury (AKI) and chronic kidney disease (CKD). Prospective cohort studies of AKI have shown the estimated incidence of DIN to be between 14 and 26% in adult populations [1,2]. Furthermore, 16% of hospitalised AKI cases in paediatrics can be attributed to nephrotoxic drugs [3]. AKI arising from DIN often results in the development of progressive CKD or end-stage kidney disease, both associated with a high mortality rate [4]. Experimental testing of all potential nephrotoxic drugs is not possible, which makes computer analyses of available data in order to preliminarily evaluate substances of interest for nephrotoxic activity a very attractive alternative to experiments.

The ability to discern nephrotoxic structures during the initial stages of drug development presents an opportunity to improve patient health outcomes. However, the mechanisms of DIN are intricate and can vary between drug classes. These distinctions are typically generalised based on the histological component of the kidney that is primarily affected. Several processes can cause nephrotoxicity involving diverse segments, such as glomerular damage, glomerulonephritis and interstitial nephritis, renal tubular injury and cytotoxicity, leading to necrosis and tubular obstructions due to drug-induced crystallopathy [5,6]. Drug metabolism also plays a role in toxicant bioactivation, by forming either proximate toxic metabolites or stable reactive intermediates. This process can occur during phase I reactions, when new or modified functional groups are formed or cleaved, often followed by phase II reactions, involving conjugation with an endogenous substance, as, for example, seen with cadmium or aristocholic acid also coupling the liver to the kidney [7,8]. An accurate prediction and evaluation of nephrotoxicity during the initial stages of drug development is necessary to identify new therapeutics. Currently, preclinical testing of compounds relies heavily on in vivo systemic toxicity animal studies to examine their effects throughout varying dosage regimens over different durations. However, the establishment, processing and analysis of kidney histopathology samples acquired from these studies are expensive, time-consuming and insufficient for screening large numbers of compounds, and also raise several ethical issues concerning animal welfare [9,10]. Furthermore, animal models may not accurately predict human renal drug handling [11]. In contrast, numerous cell-based in vitro assays have been developed towards the early identification of toxicity traits for potential drug candidates, yet such studies have been limited due to the number and type of cells employed, the simulated microenvironments and methods of drug exposure [12,13]. Despite the advancements in increasingly predictive in vitro models, the development of improved in silico approaches is of paramount importance [14]. Successful assimilation of in silico models would permit the adoption of endpoints from the clinical and regulatory setting. These computational tools could better utilise high quality ‘known’ data points to form predictions based on biological complexities and experimental scalability, both essential for producing meaningful and robust datasets that permit improved integration of predictive in silico models.

Experimental and clinical testing of drugs of their toxic potential are expensive and time-consuming actions. Computational analyses of available databases on drug-induced toxicity is an attractive alternative. Some models have been developed on nephrotoxicity, addressing general nephrotoxicity and/or specific endpoints, such as tubular necrosis [15,16,17,18,19,20,21]. However, these studies focus on different perspectives and relate, for instance, to some particular categories of substances (e.g., andrographolide derivatives, traditional medicines and drugs) based on different exposure scenarios, and address endpoints that do not overlap. Thus, more efforts have to be made to produce better predictive models for nephrotoxicity.

**Figure 1:**
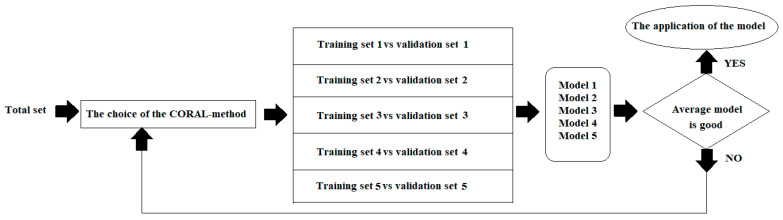
The general scheme of the study using CORAL software.

The use of CORAL software (http://www.insilico.eu/coral, accessed on 15 March 2023) is one of the ways to solve this task. The most common approach to computational modelling is to calculate molecular descriptors first, and then, using them, to develop an in silico model. Compared to the traditional approach, CORAL has the advantage that it does not require the calculation of the molecular descriptors; instead, it simply uses the molecular formula of the potential toxicant represented as SMILES [22]. The purpose of this study was to evaluate the possible use of CORAL to develop better, simpler models to accurately predict nephrotoxicity. The general scheme of the study is represented in Figure 1.

## 2. Materials and Methods

Categorical data on drug induced nephrotoxicity (n = 565) were taken from the literature [19]. Table 1 contains an overview of the selection of reported nephrotoxic drugs and their toxicity profiles.

The endpoint addressed contained 565 diverse chemical structures, including real world data on 287 nephrotoxic drugs in humans and 278 non-nephrotoxic approved drugs. These compounds were randomly distributed in the active training (≈25%), passive training (≈25%), calibration (≈25%) and validation sets (≈25%). Each of these sets has a special task: (i) the active training set provides the foundation of the model, i.e., compounds of this set are used for building up the predictive model; (ii) the passive training set is the inspector of the model, i.e., compounds of this set are used to verify whether the model is satisfactory for substances which are absent in the active training set; (iii) the aim of the calibration set is to detect the start of the overtraining; and (iv) the validation set is used for the final validation of the predictive potential of the model, using substances that were not used to develop the model.

### 2.1. Optimal SMILES-Based Descriptors

Two optimal descriptors calculated with attributes of SMILES are examined here:

(1) $$DCW1\left(T,N^{}\right)=\sum_{k=1}^{NA}CW\left(S_{k}\right)+\sum_{k=1}^{NA-1}CW\left({SS}_{k}\right)+\sum_{k=1}^{NA-2}CW\left({SSS}_{k}\right)$$

(2) $$DCW2\left(T,N^{}\right)=CW\left(AAP\right)+\sum_{k=1}^{NA}CW\left(S_{k}\right)+\sum_{k=1}^{NA-1}CW\left({SS}_{k}\right)+\sum_{k=1}^{NA-2}CW\left({SSS}_{k}\right)$$

Two descriptors which are the sum of the so-called correlation weights are examined here. The correlation weights are coefficients calculated with the Monte Carlo method. The second version of the optimal SMILES-based descriptor was calculated with atoms pairs proportions (APP) correlation weights [23]. 

Using descriptor values, one can calculate y (category qualifier) using the so-called semi-correlation technique [24]:

(3) $$y=C_{0}+C_{1}\times DCW\left(T,N\right)$$

and then define the category of a substance:

(4) $$Category\left(SMILES\right)=\left\{\begin{array}{cc} active & if,y\geq 0.5\\ inactive & if,y<0.5 \end{array}\right.$$

*S_k_* is a SMILES atom, i.e., one symbol (‘C’, ‘O’, ‘N’) or group of symbols which cannot be examined separately (‘Cl’, ‘Br’, ‘%11). *SS_k_* and *SSS_k_* combines two and three SMILES atoms, respectively. CW(*S_k_*), CW(*SS_k_*) and CW(*SSS_k_*) are the correlation weights of the above SMILES fragments. 

### 2.2. Monte Carlo Optimisation

The optimal SMILES-based descriptor requires numerical data on the correlation weights. Monte Carlo optimisation is a tool to calculate these correlation weights. Here, two target functions for the Monte Carlo optimisation were examined:

(5) $${TF}_{1}=r_{AT}+r_{PT}-\left|r_{AT}-r_{PT}\right|\times 0.1$$

(6) $${TF}_{2}=r_{AT}+r_{PT}-\left|r_{AT}-r_{PT}\right|\times 0.1+IIC\times 0.5$$

where $r_{AT}$ and $r_{PT}$ are the correlation coefficients between the observed and predicted endpoint for the active training set and passive training set, respectively. *IIC_C_* is the index of ideality of correlation calculated with data on the calibration set as follows [25]:

(7) $${IIC}_{}=r_{C}\frac{min{(}^{-}{MAE}_{C}{,}^{+}{MAE}_{C})}{max{(}^{-}{MAE}_{C}{,}^{+}{MAE}_{C})}$$

(8) $$\min\left(x,y\right)=\left\{\begin{array}{c} x,if\;x<y\\ y,otherwise \end{array}\right.$$

(9) $$max\left(x,y\right)=\left\{\begin{array}{c} x,if\;x>y\\ y,otherwise \end{array}\right.$$

(10) MAEC−=1N−∑∆k,N is the number of∆k−<0

(11) MAEC+=1N+∑∆k,N is the number of∆k+≥0

(12) $$\Delta_{k}={observed}_{k}-{calculated}_{k}$$

where the ${observed}_{k}$ and ${calculated}_{k}$ are the observed and calculated values of endpoint, respectively. 

The calculations were carried out in fifteen epochs. An epoch is a step-by-step modification of all correlation weights. The sequences of these modifications are random and different for each epoch.

## 3. Results

We tried different approaches to develop in silico models. Table 2 contains the statistical quality of model 1 for DIN obtained with *DCW1(T,N)* and the Monte Carlo optimisation with target function calculated with Equation (5). This approach is the classical CORAL approach. Table 3 contains the statistical quality of model 2 for the endpoint observed in the case of *DCW2(T,N)* and target function calculated with Equation (5). In this case, a more sophisticated algorithm is used, adopting Equation (2) and not Equation (1). Comparing the results shown in Table 2 and Table 3 on the total set of compounds, it is noted that all the statistical parameters are better in Table 3; thus, the use of Equation (2) is preferable. Table 4 contains the statistical quality of model 3 observed in the case of *DCW2(T,N)* and the target function calculated with Equation (6). In this case, we applied a further improvement in the algorithm, using the index of ideality of correlation. Considering the results for the total set, we observe that all statistical parameters are better (the value for sensitivity remains the same). There are other preferable aspects that can be interpreted from Table 4. With the previous models, comparing Table 2, Table 3 and Table 4, it is possible to observe a larger spread of values obtained for the different sets. In some cases, the values are very high, but then the results are worst for other sets. This indicates a lower robustness of the model compared to the results of the model in Table 4. For instance, the Matthews correlation coefficient (*MCC*) values range from 0.28 to 0.71 in Table 2, from 0.27 to 0.94 in Table 3 and from 0.61 to 0.89 in Table 4. Furthermore, considering Table 2 and Table 3, the results of the validation set were not always high. Conversely, the results in Table 4 are the highest of the three tables for all the statistical parameters. The results on the validation set are those obtained once the model is complete, and are used to predict a set of substances never used in the steps of model building. Thus, this value can indicate the expected performance when the model is used for new substances.

**Figure 2:**
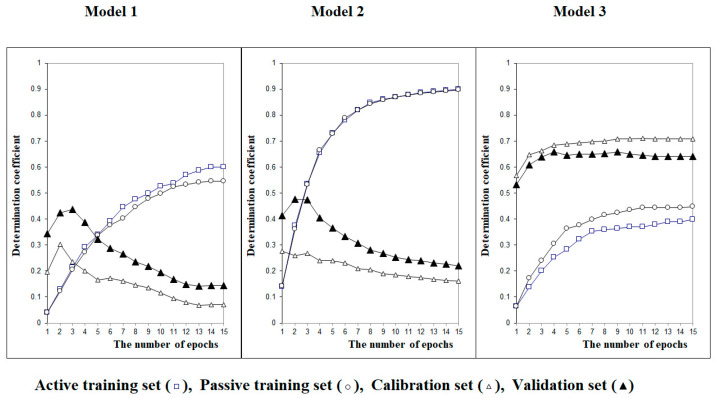
The evolution of the Monte Carlo optimisation for building up models 1, 2 and 3 with different numbers of epochs.

Figure 2 demonstrates the difference between models 1, 2 and 3. Again, on visual inspection, it is clear that the spread of values is smaller using model 3, and the values are higher for the validation set. Furthermore, we can see 15 epochs are sufficient in our case to reach the plateau, after which, no further significant improvement is obtained.

## 4. Discussion

Alternative (without the use of animals) methods for testing chemicals involve an entire arsenal of tools developed for QSAR analyses. This includes gradient machine learning methods [26], artificial neural networks and support vector machines [27]. An important component of the modelling of toxicity to various organs is the involvement of the toxicokinetic ideas [28]. Nevertheless, drug-induced nephrotoxicity remains a common problem with exposure to medications and diagnostic agents [29].

Incorporating data on drug molecular operating environment (MOE) descriptors allowed the construction of highly predictive models, as characterised by values of sensitivity of 0.87, specificity of 0.87 and MCC of 0.74. 

Thus, the described approach results in a model comparable with related models in the literature [19]. Compared to the models in the literature, the advantage of the CORAL model is its simplicity. There is no need to calculate chemical descriptors, and all the necessary chemical information used by the algorithm is contained within the SMILES structure. The traditional approach calculates molecular descriptors from the chemical structure and then applies the algorithm to build up the model. Our simplified approach, instead, has a great advantage not only in the development phase of the model, but also in its use. Indeed, the approach is much more direct and reproducible (Supplementary materials, Tables S1–S3). 

We will implement this model for nephrotoxicity within the VEGAHUB platform (www.vegahub.eu, accessed on 15 March 2023), and in this way the model will be freely and openly available. The availability of this model will help industry to screen new substances they want to develop, anticipating possible critical effects. It must be noted that the model is suitable only if the compounds fall within the applicability domain of the model. This is the usual limitation of any in silico model, since the model learns from the available experimental data. Modern software tools, such as the one we have developed, can cope with this considering the chemical structure of the substance to be evaluated. In this particular case, the model refers to pharmaceutical substances. Since there are multiple pharmaceutical classes, it is likely that the model does not cover all pharmaceuticals, and for this the implementation within VEGAHUB will be useful, since in this way the applicability domain will be measured automatically. We are also developing further models for kidney toxicity at different levels, addressing adverse outcome pathways and the no observed adverse effect level.

## 5. Conclusions

The best predictive potential was observed for model 3, which gave an accuracy of 0.87, a specificity of 0.91 and a sensitivity of 0.83; thus, the model is quite balanced. This model was obtained using an advanced approach, with the optimal SMILES-based descriptor calculated with correlation weights of the APP vector and obtained with a Monte Carlo optimisation based on the target function calculated with Equation (6), i.e., with application of the index of ideality of correlation. Thus, (i) the suggested APP vector correlated with the nephrotoxicity and (ii) the index of ideality of correlation as a measure of the predictive potential of a model improves the model performance and is an effective component of the target function for the Monte Carlo optimisation used to develop the model.

In the final analysis, we present a novel deployment of CORAL software to predict drug-induced nephrotoxicity. Our model was optimised using previously published and widely accessible SMILES data and is comparable to similar techniques for classifying drugs as toxic or non-toxic. Thus, this in silico model may prove useful for predicting nephrotoxicity for novel substances as a stand-alone method or as part of an integrated approach at a much earlier stage of clinical development, therefore saving resources in terms of animal models, human resources and indeed sponsor investment. 

## Figures and Tables

**Table 1:** Frequently reported nephrotoxic chemicals and their toxicity profiles.

Compounds	Renal Toxicity Profile										
	AIN *	ATN	Altered Haemodynamics	CIN	Crystal Nephropathy	Glomerulo-Nephritis	Inflammation	PTCT	PTI	Thrombotic Micropathy	Tubular Obstruction
Acyclovir	X				X		X		X		X
Amphotericin B		X	X					X			
Ampicillin	X				X	X	X				
Ceftriaxone	X					X	X				
Cidofovir		X						X	X		
Ciprofloxacin	X				X						
Cisplatin		X		X			X	X	X		
Cyclosporine		X	X	X						X	
Foscarnet		X			X			X			X
Ganciclovir					X						X
Gentamicin		X						X			
Ibuprofen	X		X	X		X	X				
Indinavir	X				X		X				X
Mannitol								X			
Methotrexate		X			X						X
Naproxen	X	X									
Penicillin	X	X				X	X				
Rifampicin						X	X				
Streptozocin		X						X			
Sulphadiazine					X		X		X		X
Sulphamethoxazole	X				X		X				X
Tacrolimus		X	X							X	
Tenofovir		X						X			
Triamterene	X				X						
Vancomycin	X	X			X		X				X

(*) AIN = acute interstitial nephritis, ATN = acute tubular necrosis, CIN = chronic interstitial nepthritis, PTCT = proximal tubular cell toxicity, PTI = proximal tubular injury, RAS = renal artery stenosis.

**Table 2:** The statistical quality of model observed for the first optimal descriptor with the first version of the Monte Carlo optimisation of the correlation weights.

Set	Set	Observed Classification Quality					The SAR Statistics			
		TP	TN	FP	FN	N	Sensitivity	Specificity	Accuracy	MCC
Training	A	55	62	10	10	137	0.85	0.86	0.85	0.71
	P	73	51	6	11	141	0.87	0.89	0.88	0.75
	C	48	45	27	25	145	0.66	0.63	0.64	0.28
Validation		46	51	26	19	142	0.71	0.66	0.68	0.37
Total set		222	209	69	65	565	0.77	0.75	0.76	0.53

**Table 3:** The statistical quality of model observed for the second optimal descriptor with the first version of the Monte Carlo optimisation of the correlation weights.

Set	Set	Observed Classification Quality					The SAR Statistics			
		TP	TN	FP	FN	N	Sensitivity	Specificity	Accuracy	MCC
Training	A	63	70	2	2	137	0.97	0.97	0.97	0.94
	P	82	54	3	2	141	0.98	0.95	0.96	0.93
	C	48	44	28	25	145	0.66	0.61	0.63	0.27
Validation		44	60	17	21	142	0.68	0.78	0.73	0.46
Total set		237	228	50	50	565	0.83	0.82	0.82	0.65

**Table 4:** The statistical quality of model observed for the third optimal descriptor with the second version of the Monte Carlo optimisation of the correlation weights.

Mission	Set	Observed Classification Quality					The SAR Statistics			
		TP	TN	FP	FN	N	Sensitivity	Specificity	Accuracy	MCC
Training	A	47	63	9	18	137	0.72	0.88	0.80	0.61
	P	61	52	5	23	141	0.73	0.91	0.80	0.63
	C	69	68	4	4	145	0.95	0.94	0.94	0.89
Validation	V	60	70	7	5	142	0.92	0.91	0.92	0.83
Total set		237	253	25	50	565	0.83	0.91	0.87	0.74

## Supplementary Materials

The following are available online at https://www.mdpi.com/article/10.3390/toxics11040293/s1, Tables S1–S3 contain technical details on the splits 1–3, respectively.

## Abbreviations

**Abbreviation**	**Comments**
QSAR	Quantitative stricture–activity relationship
DIN	Drug-induced nephrotoxicity
AIN	Acute interstitial nephritis
ATN	Acute tubular necrosis
SMILES	Simplified molecular input-line entry system
CIN	Chronic interstitial nephritis
PTCT	Proximal tubular cell toxicity
PTI	Proximal tubular injury
RAS	Renal artery stenosis
IIC	Index of ideality of correlation
DCW	Descriptor of correlation weights
TP	True positive
TN	True negative
FP	False positive
FN	False negative
MCC	Matthews correlation coefficient
A	Active training set
P	Passive training set
C	Calibration set
V	Validation set
